# Ziconotide and psychosis: from a case report to a scoping review

**DOI:** 10.3389/fnmol.2024.1412855

**Published:** 2024-10-16

**Authors:** Marc Peraire, Rita Gimeno-Vergara, Jennifer Pick-Martin, Mireia Boscá, Iván Echeverria

**Affiliations:** ^1^TXP Research Group, Universidad Cardenal Herrera-CEU, CEU Universities, Castellón de la Plana, Spain; ^2^Department of Mental Health, Consorcio Hospitalario Provincial de Castellón, Castellón de la Plana, Spain; ^3^Department of Psychiatry, Laboratory for Neuropsychiatry and Neuromodulation, Massachusetts General Hospital, Harvard Medical School, Boston, MA, United States

**Keywords:** ziconotide, psychosis, Cav2.2, GABA, glutamate, dopamine, scoping review, neuropsychiatric symptoms

## Abstract

Ziconotide is a non-opioid analgesic that acts on N-type voltage-gated calcium channels. Despite its proven effectiveness in pain treatment, it can induce neuropsychiatric symptoms. The aim of this article is to present a case of psychosis secondary to ziconotide and to explore the variety of neuropsychiatric symptoms it produces, exploring the relationship between these symptoms and the mechanism of action of ziconotide. For this purpose, a clinical case is presented as well as a scoping review of other cases published in the scientific literature. A search on Web of Science, Pubmed and Embase databases was performed on December 11, 2023, following the criteria of the PRISMA-ScR Statement. The clinical case presented shows the variety of neuropsychiatric symptomatology that ziconotide can cause in the same patient. On the other hand, 13 papers were retrieved from the scoping review (9 case reports, 4 case series), which included 21 cases of patients treated with ziconotide who presented adverse effects ranging from psychotic symptoms to delirium. In conclusion, the variety of neuropsychiatric symptoms derived from ziconotide could be related to the blockade of N-type voltage-gated calcium channels in glutamatergic and GABAergic neurons, in turn affecting dopaminergic pathways.

## 1 Introduction

Ziconotide is a non-opioid analgesic that has contributed to a paradigm shift in pain treatment. It is a synthetic derivative of *ω*-Conotoxin MVIIA found in the venom of the marine snail Conus magus. The molecule consists of 25 amino acids containing 6 cysteine residues linked by three disulfide bridges that fold the structure and produce the characteristic 3D structure that is critical for its mechanism of action based on the selective blockade of N-type voltage-gated calcium channels (Cav2.2) (Brookes et al., 2017). These channels play a crucial role in the release of neurotransmitters, such as glutamate and substance P, responsible for pain transmission (Zamponi et al., 2015; Moore and McCrory, 2017).

Several cases of ziconotide-treated patients experiencing adverse effects, particularly neuropsychiatric symptoms, have been reported in the literature, including confusion, disorientation, decreased alertness, somnolence, drowsiness, hallucinations or other changes in perception and mood. These symptoms were already known from early clinical trials, and can last for as long as ziconotide is administered and for up to 2 weeks after discontinuation, although in some cases they may persist (Smith and Deer, 2009).

Indeed, there are some patient profiles that are more prone to experience these side effects, such as those with pre-existing psychiatric disorders (Smith and Deer, 2009). However, since this drug is usually prescribed by non-mental healthcare professionals, psychiatric symptoms are described briefly and vaguely as ‘hallucinations’ or ‘confusion.’ Thus, such a non-specific symptoms could be compatible with diagnoses as diverse as delirium or psychosis.

Despite the knowledge of the mechanism of action of the ziconotide and its association with neuropsychiatric adverse effects, there is a gap in the literature regarding the underlying physiological mechanisms. Although some hypotheses suggest that psychotic symptoms may be favored by the blockade of Cav2.2 in the prefrontal cortex and its relationship with dopaminergic neurotransmission (Burdge et al., 2018), no work has explored a specific pathway that may be involved in the appearance of these symptoms.

Therefore, our aim is to describe, from a novel psychiatric perspective, a ziconotide-induced psychotic episode with a wide range of neuropsychiatric symptoms. At the same time, this case report allowed a scoping review to analyze the clinical diversity of other published cases and to hypothesize about possible specific pathways associated with these adverse symptoms.

## 2 Methods

This case report was selected and described by the same mental health professionals that attended the patient. For this purpose, consent was requested from the patient herself and her relatives to obtain data from the clinical history.

Subsequently, a scoping review was conducted following the criteria of the PRISMA-ScR Statement, which was registered in the Open Science Framework (doi: 10.17605/OSF.IO/7C8RQ). A systematic review was completed on December 11, 2023, in the Web of Science, Pubmed and Embase databases. This combination was used because it ensured a recall of almost 96% of the papers (Bramer et al., 2017).

**Figure 1:**
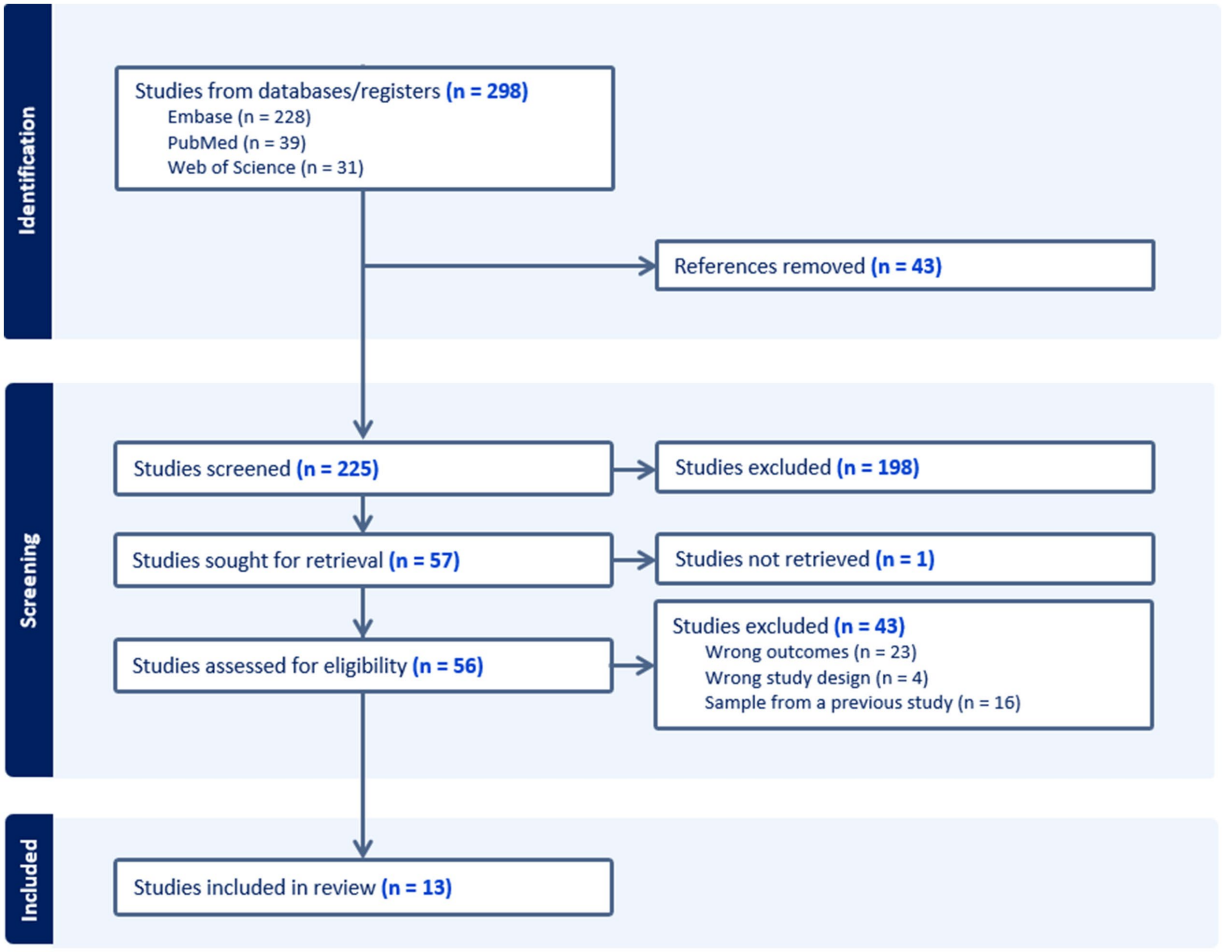
PRISMA flow diagram of study selection.

The terms chosen for the search were: ziconotide AND (psycho* OR delirium OR hallucination OR delusion). No filter was added, but the field code /exp was added to these terms in Embase to search for the related narrower or child terms. The references obtained (Figure 1) were entered into the systematic review management software COVIDENCE. The PICOS (participants, intervention, context, outcomes, and study design) framework was used to establish eligibility criteria. Two reviewers analyzed independently the results obtained according to the inclusion and exclusion criteria, and the resulting discrepancies were resolved by a third reviewer.

As inclusion criteria, case reports or case series were considered, whether in the form of an article, posters or oral communication, which included patients treated with ziconotide and who had presented psychosis, hallucinations, delusions or delirium. As exclusion criteria, we rejected those studies in which the psychopathology could not be clearly attributed to ziconotide, considering that the use of opioids can lead to confusional or even psychotic symptoms; studies that only provide the prevalence of neuropsychiatric adverse effects, but do not examine in depth the course of the disease itself; those cases in which there was a history of psychiatric disorders; and those studies whose language was not English or Spanish. The results of the studies obtained were grouped according to the clinical presentation and/or reported symptomatology (hallucinations, delirium, psychosis or delirium).

## 3 Results

### 3.1 Case presentation

The patient was a 54-year-old woman who was admitted to the psychiatric emergency room for auditory verbal hallucinations that began a few weeks earlier. Concretely, she felt that her son and his partner were talking about her and insulting her through speakers.

Her medical history included hypothyroidism and a thoracic sarcomatoid mesothelioma diagnosed in November 2010, with multiple recurrences in the following 12 years and secondary chronic refractory pain since 2011. At the time of her consultation in the psychiatric emergency room (2023), the patient was suffering from radicular syndrome under palliative treatment with intrathecal pump of morphine 2% (19 mL) and ziconotide 100 μg/day, she had fentanyl 100 μg rescues every 72 h. The rest of her usual treatment included lormetazepam 2 mg at night, lacosamide 200 mg every 12 h, levothyroxine 50 μg every morning and metamizole 575 mg on demand.

Regarding her psychiatric history, she has suffered from dysthymia since 2006 and had been treated with escitalopram 10 mg and alprazolam 0.5 mg until 2013, when she discontinued them.

On initial psychiatric evaluation, the patient was conscious but disoriented as to time, place, and person. Her appearance was cachectic, and her attitude was suspicious and distressed. Slow thinking was noted as well as incoherent and derailed speech evidencing delusions of prejudice toward his son and his partner. The patient also described self-references, being spied on through electronic devices, and false recognition phenomena. Moreover, she admitted to having complex visual hallucinations (human figures), gustatory (dysgeusia) and olfactory (parosmia). The patient also had a long history of depressed mood in response to the prolonged oncologic process, with passive suicidal ideation, hyporexia, and insomnia.

Complementary tests, brain CT and chest X-ray showed no significant findings of oncologic progression. Toxicological analysis was positive for morphine and benzodiazepines, compatible with her usual medication. Family members reported that the only significant change prior to her current situation was the adjustment of analgesic medication 8 days before due to inadequate pain control, increasing ziconotide from 0.2 µg/day to a total dose of 7.5 µg/day.

The patient was admitted to the psychiatric ward, where she presented gait instability, tremor, and fluctuation of temporal and spatial orientation during the first days of admission. She maintained a certain level of uneasiness and suspicion with her relatives and healthcare professionals, introducing them to her delusions of harm, and having episodes of hostility that required the administration of intramuscular haloperidol and five-point mechanical restraint to ensure her own safety. Olanzapine (15 mg/day) was initiated, but the patient continued with delusional, hallucinatory, false recognitions and mumbling speech.

Considering the complex clinical picture, the possibility of organic psychosis or psychosis induced by some of her usual treatments was suggested, as this picture was compatible with the adverse effects of ziconotide. Given the clinical suspicion, a progressive reduction of intrathecal ziconotide was agreed with the Pain Management Unit until discontinuation.

As the dose of ziconotide was reduced, the psychotic symptoms disappeared, until they completely resolved 48 hours after drug withdrawal. After 12 days of hospitalization, the condition was classified as a ziconotide-induced psychotic episode. The dose of olanzapine was reduced to 5 mg and the patient was referred to the Pain Unit for pain control, where the antipsychotic was eventually discontinued without recurrence of delusional symptoms.

### 3.2 Scoping review

A total of 13 papers (9 case reports, 4 case series) were included in this scoping review (Burdge et al., 2018; Su et al., 2021; Phan and Waldfogel, 2015; Whitlow et al., 2015; Abdelsayed et al., 2022; Stewart and Sarria, 2014; Kapural et al., 2009; Ayuga-Loro et al., 2011; Obafemi and Roth, 2013; Grajny et al., 2020; de la Calle Gil et al., 2015; Levin et al., 2002; Penn and Paice, 2000). They included 30 cases of patients with ziconotide who presented adverse effects, 21 of which consisted of neuropsychiatric symptoms (Table 1).

**Table 1:** Summary table of ziconotide case report characteristics.

Author (year)	Country	Patient characteristics	Ziconotide peak dose /adverse effect dose	Neuropsychiatric symptoms	Management and treatment
Burdge et al. (2018)	United States	Male, 49 yo Chronic back pain after motor vehicle accident (spinal fusion surgeries) No previous neuropsychiatric history	14 mcg/d	Paranoia, auditory hallucinations, racing thoughts, pressure speech, labile affect and agitation	Ziconotide was reduced to 7 mcg/day, discontinued after 5 days, and risperidone 1 mg was added. After 10 days, the patient was discharged with risperidone 1 mg at bedtime
Su et al. (2021)	United States	Male, 60 yo Chronic back pain following a fall (L4 and L5 laminectomy) No previous neuropsychiatric history	7.37 mcg/d	Paranoid delusions, irrational beliefs of infestation, auditory, tactile and visual hallucinations, soliloquies, intermittently unintelligible speech and overvaluation of abilities	Ziconotide was reduced to 1.2 mcg/d and risperidone 0.5 mg/12 h was started. After 8 days, ziconotide was reduced to 0.158 mcg/d and risperidone was increased to 2 mg/12 h. The patient’s psychiatric adverse effects improved and he was discharged with risperidone 2 mg/12 h
Phan and Waldfogel (2015)	United States	Female, 49 yo Complex regional pain syndrome related to a previous spinal injury (cervical fusion, spinal cord stimulation implantation) History of anxiety and depression	4.9 mcg/d	Paranoia, harm delusions, psychotic fear and hallucinations	Ziconotide was discontinued and risperidone 0.5 mg was started, with resolution of hallucinations and delusions in the first few hours
Whitlow et al. (2015)	United States	Female, 55 yo Stage IV endometrial cancer being treated with oral chemotherapy History of anxiety and depression	2.53 mcg/d	Fear (due to suspected poisoning), auditory and visual hallucinations, overestimation of abilities, recent memory impairment and agitation	Paliperidone 6 mg was started. After 7 days, a monthly injection of 234 mg paliperidone was started. Symptoms persisted over the next few weeks as there were no professionals available to handle the intrathecal pump
Abdelsayed et al. (2022)	United States	Male, 60 yo Refractory back pain No history of psychosis	7.32 mcg/d	Paranoia, auditory and visual hallucinations	Ziconotide was reduced daily and neuropsychiatric side effects disappeared
Stewart and Sarria (2014)	United States	Female, 37 yo Spinocerebellar ataxia and peripheral neuropathy	10.08 mcg/d	Psychosis	Ziconotide was discontinued and symptoms resolved. After 2 months, it was reintroduced due to failure of pain control, using a flexible dosing schedule and reaching a final dose of 11,437 mcg/d. No neuropsychiatric adverse effects returned
Kapural et al. (2009)	United States	Case 3: Female, 16 yo Complex regional pain syndrome (CRPS) type I	3.5 mcg/d	Auditory hallucinations	Ziconotide was reduced to 2.5 mcg/d
		Case 4: Male, 35 yo Complex regional pain syndrome (CRPS) type I	6.48 mcg/d	Hallucinations and difficulty thinking	Ziconotide was reduced to 5.9 mcg/d and adverse effects resolved within 48 h
		Case 5: Female, 44 yo Complex regional pain syndrome (CRPS) type I	10.8 mcg/d	Visual hallucinations and cognitive impairment	Ziconotide was temporarily discontinued and the adverse effects resolved in 3 days. The pump was restarted 13 days later (5.4 mcg/d), causing panic attacks, so the dose was reduced to 4.2 mcg/d. Benzodiazepines and antipsychotics were added
		Case 6: Male, 34 yo Complex regional pain syndrome (CRPS) type I	13.44 mcg/d	Psychotic delusions, hallucinations, lack of concentration, speech difficulties, memory loss	Ziconotide was withdrawn and haloperidol and risperidone were started. Symptoms resolved in 9 days
		Case 7: Male, 52 yo Complex regional pain syndrome (CRPS) type I	11.78 mcg/d	Olfactory hallucinations, anxiety, and agitation	Ziconotide was reduced to 8.5 mcg/d
Ayuga-Loro et al. (2011)	Spain	Female, 66 yo Spinal cord injury at T12 secondary to disc prolapse after decompressive laminectomy for canal stenosis. History of depression	2.14 mcg/d	Delusional ideas of suspicion and persecution, accelerated speech and disinhibition	Ziconotide was withdrawn. Olanzapine was introduced
Obafemi and Roth (2013)	United States	Male, 37 yo Idiopathic small fiber peripheral neuropathy No history of psychosis	1.5 mcg/d	Hallucination and confusion	Ziconotide was reduced to 0.0353 mcg/day and adverse effects resolved
Grajny et al. (2020)	United States	Case 1: Female, 64 yo Spinal hemangioblastoma (resected)	6 mcg/d	Auditory hallucinations and delirium	Ziconotide was reduced to 0.25 mcg/day. Adverse effects improved but did not resolve completely
		Case 3: Male, 70 yo Cervical spondylosis History of depression	3.6 mcg/d	Hallucinations and disorientation	Ziconotide was reduced with improvement in symptoms, but was discontinued after recurrent hallucinations
de la Calle Gil et al. (2015)	Spain	Case 4: Female, 58 yo Metastatic rectal cancer and neuropathic pain	0.6 mcg/d	Neuropsychiatric disturbance	Ziconotide was withdrawn
		Case 5: Male, 63 yo Advanced colon cancer and neuropathic pain	5 mcg/d	Confusion and delirium	Ziconotide was discontinued. Due to lack of pain control, it was reintroduced, reaching a final dose of 4 mcg/day with no adverse effects
Levin et al. (2002)	United States	Male, 38 yo Painful glove-and-stocking peripheral neuropathy of unknown etiology	15.6 mcg/d	Agitated delirium	Ziconotide was withdrawn, haloperidol was started at up to 25 mg/h iv, and valproate was added. Electroconvulsive therapy was required for complete resolution of symptoms
Penn and Paice (2000)	United States	Case 1: Male, 47 yo Bladder cancer	14.4 mcg/d	Auditory and visual hallucinations and confusion	Ziconotide was withdrawn and reintroduced after 24 h due to lack of pain control. After several reductions and persistence of pain and non-neuropsychiatric adverse effects, the patient decided to discontinue ziconotide
		Case 2: Male, 62 yo Multiple sclerosis	127.2 mcg/d	Paranoid hallucinations, mild confusion, disorientation, episodes of unresponsiveness and severe agitation	Ziconotide was discontinued. Due to agitation persisting for 3 weeks after withdrawal, haloperidol 3 mg/day was added with good results
		Case 3: Male, 45 yo 10 back surgeries, spondylolisthesis, and arachnoiditis	16.8 mcg/d	Confusion, extremely talkative, nervous, and agitation	Ziconotide was increased to 18 mcg/day due to failure of pain control. Eight days later, the patient experienced syncope with nonspecific ECG changes. Ziconotide was discontinued and haloperidol was added, with gradual improvement

Considering the data in the table, it can be seen that most of the studies are from the United States. There is a male predominance in the occurrence of adverse effects (13 men vs. 8 women). Among the studies that recorded psychiatric antecedents (Burdge et al., 2018; Su et al., 2021; Phan and Waldfogel, 2015; Whitlow et al., 2015; Abdelsayed et al., 2022; Ayuga-Loro et al., 2011; Obafemi and Roth, 2013; Grajny et al., 2020), half of the patients had them (Phan and Waldfogel, 2015; Whitlow et al., 2015; Ayuga-Loro et al., 2011; Grajny et al., 2020) and the other half did not (Burdge et al., 2018; Bramer et al., 2017; Abdelsayed et al., 2022; Obafemi and Roth, 2013). On the other hand, the doses at which the adverse effects occurred were highly variable, ranging from a minimum of 0.6 mcg/d (Levin et al., 2002) to a maximum of 127.2 mcg/d (Webster, 2015).

In 7 patients the presentation was described as a “psychosis” (Burdge et al., 2018; Su et al., 2021; Phan and Waldfogel, 2015; Whitlow et al., 2015; Abdelsayed et al., 2022; Stewart and Sarria, 2014; Kapural et al., 2009), with auditory hallucinations and paranoid delusions in 5 of them (Burdge et al., 2018; Su et al., 2021; Phan and Waldfogel, 2015; Whitlow et al., 2015; Abdelsayed et al., 2022), and visual hallucinations in 3 cases (Su et al., 2021; Whitlow et al., 2015; Abdelsayed et al., 2022). However, in Stewart and Sarria (2014) the symptoms of the psychotic episode were not specified and in Kapural et al. (2009) they spoke of “psychotic delusions and hallucinations.” In addition to the above, one case had psychotic (delusions of persecution, suspiciousness) and mania-like symptoms (verbosity, disinhibition) (Ayuga-Loro et al., 2011). Continuing with hallucinations, these also occurred in isolation, with one case describing auditory hallucinations, another visual hallucinations, and yet another unspecified hallucinations (Kapural et al., 2009).

Additionally, 4 patients presented delirium, either with auditory and visual hallucinatory symptoms (Obafemi and Roth, 2013), auditory only (Grajny et al., 2020), or without any psychiatric symptoms (de la Calle Gil et al., 2015; Levin et al., 2002). Although not named as such in the cases, 5 patients presented symptoms suggestive of delirium (agitation and disorientation alternating with unresponsiveness, confusion…), either with visual and auditory hallucinations (Penn and Paice, 2000), olfactory hallucinations (Kapural et al., 2009), or unspecified hallucinations (Grajny et al., 2020), or without them (Penn and Paice, 2000).

The most important strategies for the management of neuropsychiatric symptoms have been the reduction and discontinuation of ziconotide. In turn, several studies have added the use of antipsychotics, the most common being risperidone (Burdge et al., 2018; Su et al., 2021; Phan and Waldfogel, 2015; Kapural et al., 2009) and haloperidol (Kapural et al., 2009; Levin et al., 2002; Penn and Paice, 2000). Finally, electroconvulsive therapy has been used as a strategy (Levin et al., 2002).

## 4 Discussion

The case report presented is of particular interest because it shows almost the totality of the adverse effects produced by ziconotide, providing an opportunity to reflect on its action on Cav2.2, the distribution of these channels and the relationship of all this with the aforementioned adverse effects.

Previous studies have estimated that 12% of patients treated with ziconotide have hallucinations, 3% have paranoid reactions, and 1–2% have psychosis. In addition, it has also been shown to increase the incidence of suicidality, suicide attempts, and suicidal ideations. From a neurological point of view, up to 33% may present confusion and 2% delirium (Webster, 2015). As in our case report, most of the published cases present auditory hallucinations and paranoid delusions, being generally classified as psychosis. However, the clinical overlap with delirium is striking. In this sense, a larger number of cases may not have been classified as psychosis because they are not usually described by psychiatrists, leaving psychopathology and diagnostic judgment in the background. Thus, our case report presents a psychiatric perspective that enriches the debate on the adverse neuropsychiatric effects of ziconotide.

On the other hand, the non-specificity and variety of symptoms may be due to the mechanism of action of ziconotide, which would be involved in the pathophysiological pathways responsible for symptoms of psychosis and delirium. This possibility is supported by the fact that individuals with a history of psychiatric disorders are more susceptible to ziconotide-induced adverse effects (Poli and Ciaramella, 2011), as it happens in our case report, where the patient presents dysthymia of years of evolution. That common susceptibility would be in line with the Research Domain Criteria (RDoC) approach (Cuthbert and Insel, 2013).

It has been observed that cannabinoid receptor 1 (CB1R) mediated inhibition of Cav2.2 presynaptic channels is able to suppress GABA release (a phenomenon known as depolarization-induced suppression of inhibition) (Lozovaya et al., 2009; Borgan et al., 2021). As a consequence, there is an augmentation in presynaptic dopamine in areas with an increased presence of CB1R in GABAergic neurons, such as in the cortex or striatum (Borgan et al., 2021; Szabó et al., 2014), which has been associated with the presence of positive symptoms (Borgan et al., 2021) and schizophrenia itself (Fusar-Poli and Meyer-Lindenberg, 2013). In fact, partial agonism of CB1R has been shown to be capable of producing positive, negative psychotic symptoms and cognitive deficits in healthy volunteers (Borgan et al., 2021). This suggests that inhibition of Cav2.2 channels, which is the mechanism of action of ziconotide, could play a central role in the generation of psychotic symptoms. In support of this, the CACNA1B gene, which codes for Cav2.2 channels, has been shown to be associated with schizophrenia (Andrade et al., 2019). On the other hand, the CB1R-Cav2.2 binomial is also able to suppress glutamate release in the hippocampus and anterior cingulate cortex through a similar mechanism of action (depolarization-induced suppression of excitation phenomenon) (Borgan et al., 2021), which has been associated with the occurrence of delusions and hallucinations (Egerton and Stone, 2012) and the presence of depression (Moriguchi et al., 2019; Duman et al., 2019). In fact, the Cav2.2 receptor has been directly linked to the appearance of depressive symptomatology in mice (Caminski et al., 2022). Regarding suicidal ideation, it has been hypothesized to be due to the inhibition of neurotransmitter release in areas of the brain associated with mood and cognition (e.g., prefrontal and cerebral cortex) (Webster, 2015). Likewise, CACNA1B gene has also been linked to major depressive disorder and suicide (Andrade et al., 2019) and even to bipolar disorder.

Dysregulation of neurotransmitters such as dopamine, GABA and glutamate in the prefrontal cortex has also been proposed as a causal hypothesis for delirium (Webster, 2015; Maldonado, 2013), being compatible with the pathophysiological mechanism of Cav2.2. In addition, the role of glutamate in the neuropsychiatric symptomatology present in anti-NMDA encephalitis has been demonstrated (Kayser and Dalmau, 2016) and it is speculated that both GABA and glutamate may be involved in the psychotic and affective symptomatology of Kleine-Levin syndrome (Ortega-Albás et al., 2021; Hédou et al., 2022). Finally, similar psychomimetic effects have been observed with analgesic treatments such as ketamine, which is a NMDA receptor antagonist (Corlett et al., 2016; Cheng et al., 2018).

Regarding the limitations of the study, only clinical cases were included. At the same time, not all cases explained whether there was a psychiatric history, nor did they give an accurate description of the neuropsychiatric symptomatology. Moreover, in those that did, this information was not described by mental health professionals. These facts limit the ability to infer causality, to analyze in depth the elements involved in the occurrence of these adverse effects, and thus to generalize the results. Nevertheless, the control of confounding factors such as the use of opioids and the temporal coincidence between the introduction of ziconotide and the appearance of adverse effects invite further studies to elucidate the physiopathological mechanisms responsible, which could be of interest in the study of psychosis and schizophrenia.

## 5 Conclusion

Chronic pain is a subjective symptom which is difficult to measure and has an impact on people’s quality of life. At the same time, it has traditionally been underdiagnosed and undertreated. Opioids are drugs of first choice in these conditions, although they are not always effective or adequately tolerated. Moreover, the U.S. opioid crisis has highlighted the need for alternatives to opioids because of their high addictive risk. For this reason, other analgesics have been developed to solve this problem. Ziconotide is one of them, characterized by its peculiar origin and for being the first Cav2.2 channel antagonist used for severe chronic pain via intrathecal route. However, the mechanism of action could be related in processes other than analgesia, involving neurotransmitters such as dopamine, GABA and glutamate in brain areas related to the onset of neuropsychiatric symptoms.

Although these adverse effects are not common, their appearance in patients without a psychiatric history and the risk of decompensation in those with a mental disorder suggest that certain precautions should be taken when administering this drug. In this sense, clinicians should carefully evaluate individuals with pre-existing psychiatric disorders before prescribing ziconotide. Slow titration is advisable, and clinical response as well as the occurrence of adverse effects should be closely monitored (Smith and Deer, 2009). In the event of adverse symptoms, an early and individualized approach must be taken, considering discontinuation of ziconotide or combining antipsychotics (Webster, 2015).

Therefore, it is not only necessary to investigate the mechanism of action of Cav2.2 channels in the appearance of these symptoms, but also to expand the registry of cases presenting these undesired effects in order to better understand their heterogeneity and phenomenology.

## Data Availability

The raw data supporting the conclusions of this article will be made available by the authors, without undue reservation.
